# Brucella Egresses from Host Cells Exploiting Multivesicular Bodies

**DOI:** 10.1128/mbio.03338-22

**Published:** 2023-01-09

**Authors:** Juan Manuel Spera, Francisco Guaimas, Cecilia Czibener, Juan Esteban Ugalde

**Affiliations:** a Instituto de Investigaciones Biotecnológicas, Universidad Nacional de San Martín (UNSAM)-Consejo Nacional de Investigaciones Científicas y Técnicas (CONICET), Escuela de Bio y Nanotecnologías (EByN), Universidad Nacional de San Martín, San Martín, Buenos Aires, Argentina; Yale University School of Medicine

**Keywords:** *Brucella*, egress, multivesicular bodies

## Abstract

Host cell egress is a critical step in the life cycle of intracellular pathogens, especially in microbes capable of establishing chronic infections. The Gram-negative bacterium Brucella belongs to such a group of pathogens. Even though much has been done to understand how Brucella avoids killing and multiplies in its intracellular niche, the mechanism that this bacterium deploys to egress from the cell to complete its cycle has been poorly studied. In the manuscript, we quantify the kinetics of bacterial egress and show that Brucella exploits multivesicular bodies to exit host cells. For the first time, we visualized the process of egress in real time by live video microscopy and showed that a population of intracellular bacteria exit from host cells in vacuoles containing multivesicular body-like features. We observed the colocalization of Brucella with two multivesicular markers, namely, CD63 and LBPA, both during the final stages of the intracellular life cycle and in egressed bacteria. Moreover, drugs that either promote or inhibit multivesicular bodies either increased or decreased the number of extracellular bacteria, respectively. Our results strongly suggest that Brucella hijacks multivesicular bodies to exit the host cells to initiate new infection events.

## INTRODUCTION

Intracellular pathogens can invade, survive, multiply, and eventually exit from host cells. The complete life cycle implies multiple adaptations to the intracellular compartments that the pathogen encounters in the different stages of the process and involves the regulation of gene expression in the bacterium and the biochemical remodeling of the cellular compartments in which it resides. Although much has been done to understand how intracellular bacteria invade, resist degradation, and eventually establish a replication niche, there is a paucity of knowledge on how most of these pathogens egress from the cells in order to begin a new life cycle in another cell (1–3). In the case of pathogens that are able to produce chronic infections, exit processes are central to their biology. Understanding and eventually interfering with such processes could potentially turn a chronic infection into an acute one.

Brucella, a member of this group of pathogens, is an intracellular bacterium that causes brucellosis, a widespread zoonosis that affects livestock and humans, particularly in areas of endemicity (4, 5). The virulence of this pathogen is completely dependent on its capacity to replicate in host cells, a process that involves adhesion (6–8), the invasion of the cell, the evasion of phagolysosome fusion (9–11), and the establishment of an intracellular replication niche (9). These processes are highly regulated by bacterial virulence factors that modulate host cellular responses to promote microbial survival and replication (5, 12). In the case of Brucella, less is known regarding the final stages of the intracellular life cycle, including how egress from the host cell occurs. Starr et al. (13) described that after the phase of intracellular replication, Brucella induces the formation of an autophagic-like Brucella-containing-vacuole (aBCV) and egresses from the cell in a controlled way. This last stage of the intracellular life cycle is characterized by the reacquisition of the marker LAMP-1 as well as some autophagic features that occur before the bacterial egress from the infected cells and the subsequent reinfection (13).

In the manuscript, we investigate the egress dynamics of Brucella from the host cell as well as the biochemical characteristics of the vacuole that the bacterium exploits for this final stage of the intracellular life cycle. We have monitored, for the first time, the egress of Brucella in real time by video microscopy. We found that at least a subpopulation of the intracellular Brucella exits the host cell in vacuoles with multivesicular body (MVBs) features. Our results indicate that after the intracellular replication phase, the niche is disassembled, and the Brucella-containing vacuoles (BCVs) acquire LAMP-1 and at least two markers of MVBs: CD63 and LBPA. Antibiotic protection assays with extracellular bacteria that have egressed, as well as experiments inhibiting and promoting MVBs biogenesis and fusion, confirmed that Brucella exploits these structures in the last stage of the intracellular life cycle. Finally, we observed that egressed bacteria strongly contribute to the reinfection process *in vitro*.

## RESULTS

### Kinetics of the egress of Brucella from host cells.

**FIG 1:**
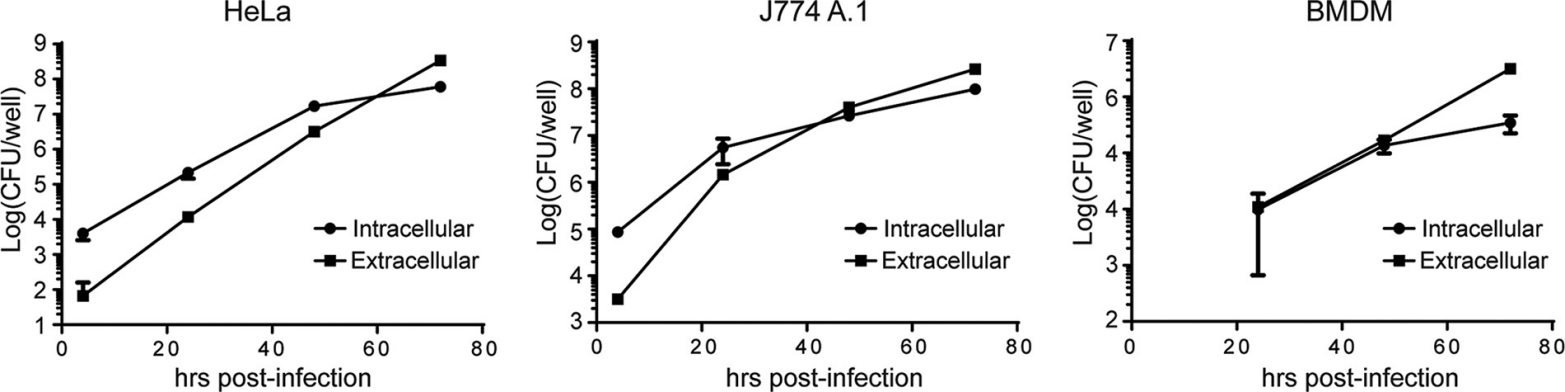
Quantification and kinetics of B. abortus egress *in vitro*. Number of intracellular (circles) and extracellular egressed bacteria (squares) in HeLa, J774 A.1, and BMDM cells at different times postinfection. The bacterial count was determined by direct plating on TSB agar plates.

**FIG 2:**
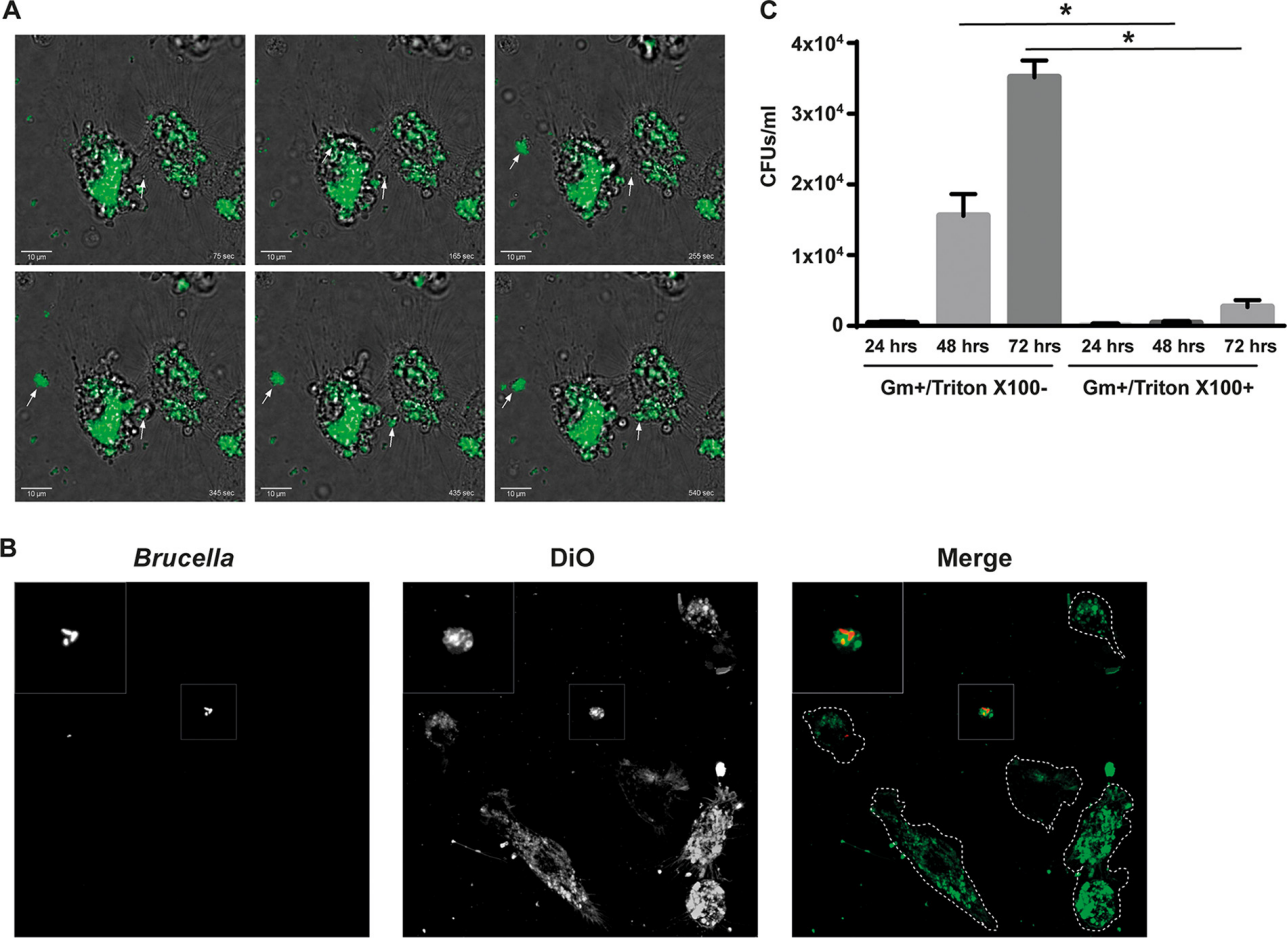
Brucella abortus exits infected cells in vesicles. (A) Images from time-lapse video microscopy, showing B. abortus DsRed (green) egressing from an infected HeLa cell (DIC). The arrows indicate a cluster of bacteria egressing in a vesicle-like structure and another cluster egressing from an infected cell and invading the neighboring one. The selected frames of merged DIC and fluorescence images are representative of the images observed between 48 and 72 h postinfection. T = 0 corresponds to 52 h postinfection. Bars: 10 μm. The corresponding time-lapse movie is shown in Supplementary Video 1. (B) Confocal images of HeLa cells infected with B. abortus DsRed at 72 h postinfection, fixed, and stained with DiO. The inset shows a cluster of extracellular bacteria. (C) Extracellular gentamicin protection assays. Egressed Brucella from infected HeLa cells, at different times postinfection, were treated for 1 h with gentamicin in the presence or absence of the detergent Triton X-100 and were quantified by direct plating on TSB-Agar. *, *P* < 0.01.

To our knowledge, the egress of Brucella from infected cells has never been visualized in real time, nor has it been quantified. To characterize the kinetics of the egress of Brucella from host cells, we performed intracellular replication assays in HeLa cells, J774 A.1 cells, and mouse bone-marrow derived macrophages. We measured, at different times postinfection, the intracellular and extracellular numbers of bacteria. As can be observed in Fig. 1, during the first 48 h of infection, the number of intracellular bacteria was equivalent to or greater than the number of extracellular bacteria, a ratio that was clearly inverted at 72 h postinfection in all analyzed cells. This observation confirms the hypothesis that, after the establishment of the intracellular replication niche (48 h postinfection), the bacteria shift to a phase in which the extracellular population (egress) outnumbers the intracellular population (trafficking and replication). To gain more detail on how the bacteria egress from the host cells, we performed live video microscopy of infected cells and monitored the egress of an RFP-tagged Brucella strain between 48 and 72 h postinfection. For this, we infected HeLa cells in a standard antibiotic protection assay. At 48 h postinfection, we washed the cells, added fresh medium without any antibiotic, and observed the infected cells under a microscope for 12 h. Fig. 2A and Supplementary Video 1 show that we were able to observe several events of egress of either individual bacteria or groups of Brucella contained in what seemed to be vesicle-like structures. In some cases, these structures egressed and moved away from the infected cells, and in others, these clusters exited one cell and invaded a neighboring one.

The data from the video microscopy experiments showed that many of the bacteria egressing from the host cells, when observed under phase contrast, showed a surrounding membrane structure. To initially evaluate whether this was the case, we infected HeLa cells with B. abortus DsRed and, at 72 h postinfection, fixed the infected cells and stained them with the lipophilic dye DiO that stains cellular membranes (18). As can be observed in Fig. 2B, we found that many of the extracellular bacteria stained positive for DiO, indicating that they were surrounded by or associated with cellular membranes. To further confirm this finding, we collected extracellular bacteria that had egressed from infected HeLa cells between 2 and 24 h, 24 and 48 h, or 48 and 72 h postinfection and treated them with gentamicin, an antibiotic that is unable to cross the eukaryotic membrane, for 1 h in the presence or absence of the detergent Triton X-100. The number of viable bacteria after the treatments was determined by direct plating on tryptic soy broth (TSB) agar. As can be observed in Fig. 2C, treatment with the detergent significantly increased the number of gentamicin-sensitive bacteria, indicating that at least part of this extracellular population was surrounded by an antibiotic-protective membrane. Fig. S1 shows that Triton X-100 does not affect the viability of Brucella.

10.1128/mbio.03338-22.1 FIG S1 B. abortus was grown in liquid culture, washed, diluted to a final optical density of 1 in PBS in the presence (0.1%) or absence of Triton X-100, and incubated for 60 min. After the incubation, the bacteria were serially diluted and plated on TSB-agar plates for 48 to 72 hrs, until colonies developed. The quantification of viable bacteria was performed by a direct count. Download FIG S1, TIF file, 1.3 MB. Copyright © 2023 Spera et al. 2023 Spera et al. https://creativecommons.org/licenses/by/4.0/ This content is distributed under the terms of the Creative Commons Attribution 4.0 International license.

### Brucella exploits multivesicular bodies for the egress.

Multivesicular bodies (MVBs) are specialized endosomes that contain intraluminal vesicles that are generated from the invagination and budding of the limiting membrane (19). In the endocytic pathway, MVBs are late endosomes whose content can be degraded through fusion with lysosomes/vacuoles or released into the extracellular space after fusion with the plasma membrane (20).

**FIG 3:**
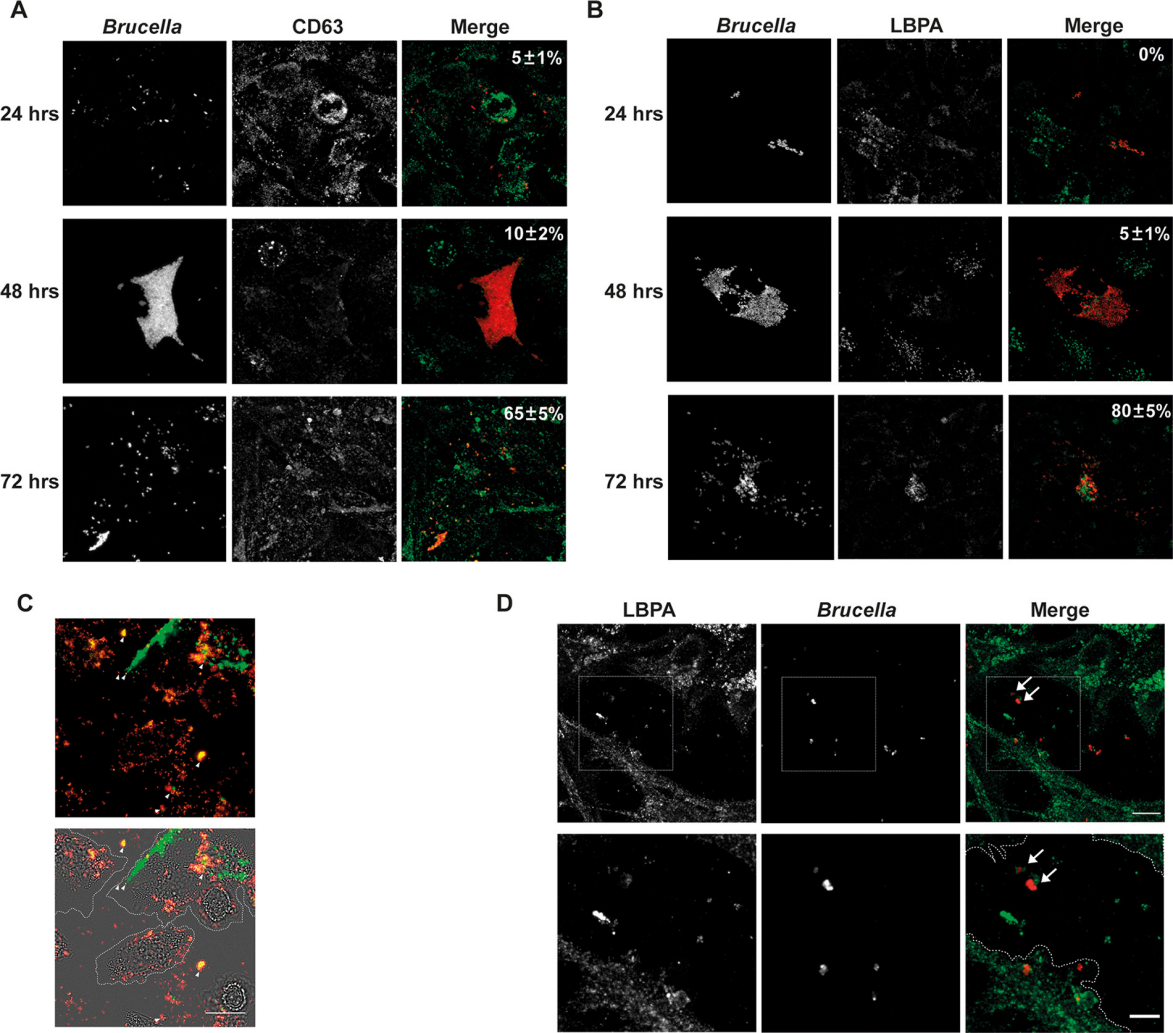
Intracellular and extracellular colocalization of B. abortus with MVBs markers. HeLa cells were either infected with B. abortus (A, B, and D) or transfected with CD63-GFP, infected with B. abortus DsRed (C), and subjected to immunofluorescence microscopy. The images show the colocalization of either intracellular bacteria (red) with CD63 (A) and with LBPA (B), or extracellular bacteria with CD63 (C) and with LBPA (D) (all in green) at different times postinfection. The dotted line indicates the cellular membrane. Bars: 10 μm.

**FIG 4:**
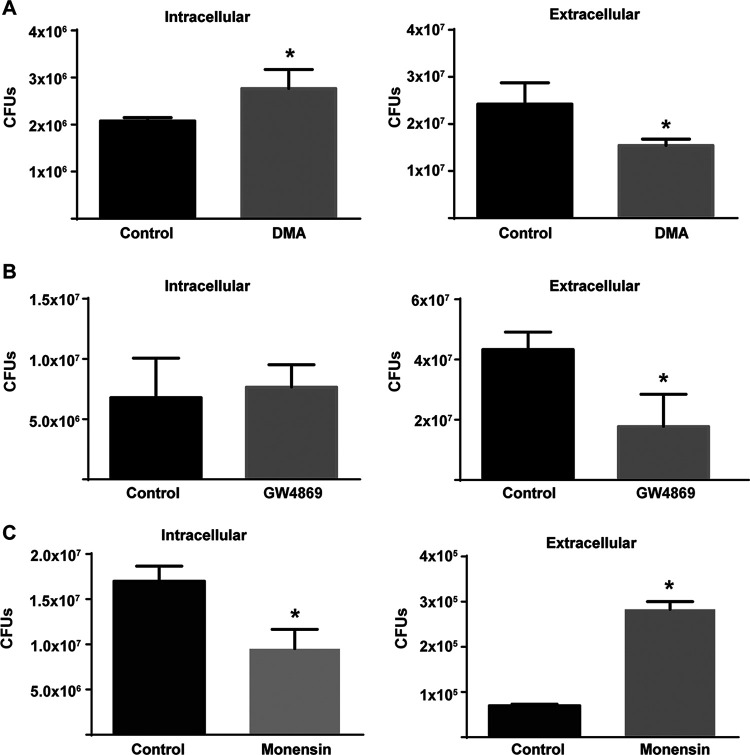
The drug manipulation of MVBs affects the egress of Brucella from infected cells. HeLa cells were infected with B. abortus and were either treated or not with the MVBs inhibitors (A) 15 nm DMA, (B) 5 μm GW4869, or (C) the MVBs promoter 7 μm monensin. The intracellular and extracellular bacteria were quantified by direct plating on TSB agar plates. *, *P* < 0.05.

Our observations of extracellular bacteria that were apparently surrounded by membranes prompted us to analyze the colocalization of these structures with two canonical markers of MVBs: CD63 (17, 21–23) and LBPA (lisobisphosphatidic acid) (17, 22–25). Figure 3A shows that at 72 h postinfection, 65 ± 5% of the BCVs acquired CD63, a marker present in only 5 ± 1% at 24 h and in 10 ± 2% of the BCVs at 48 h. To confirm the hypothesis that this BCV acquires MVB features, we analyzed the colocalization with a different marker, namely, LBPA. Figure 3B shows that, as with CD63, LBPA colocalized with 80 ± 5% of the BCVs at 72 h postinfection but was absent at 24 h, whereas only 5 ± 1% of the BCVs showed colocalization at 48 h. Similar results were obtained with the macrophagic cell line J774 A.1 (Fig. S2). Interestingly, we observed extracellular bacteria colocalizing with CD63 and LPBA (Fig. 3C and D), indicating that some extracellular egressed bacteria also present MVB markers. To further confirm that Brucella exploits MVBs for the egress phase, we performed infections and determined the efficiency of egress in the presence of drugs that inhibited or promoted the formation of MVBs. As can be observed in Fig. 4A and B, the treatment of infected cells with two inhibitors of the formation of MVBs (DMA and GW4849) (17, 26) diminished the number of extracellular bacteria between 48 and 72 h postinfection. On the contrary, treatment with monensin, a drug that promotes the biogenesis of MVBs and exocytosis (27–29), increased the number of extracellular bacteria (Fig. 4C). These results were replicated in J774 A.1 cells (Fig. S3). Taken together, these results support the hypothesis that the BCV exploits the multivesicular bodies biogenesis pathway for the egress of Brucella from infected cells.

10.1128/mbio.03338-22.2 FIG S2 Colocalization of B. abortus with MVB marker LBPA in J774 A.1 cells. J774 A.1 cells were infected with B. abortus DsRed, fixed at 24, 48, and 72 hrs postinfection, and subjected to immunofluorescence microscopy. The images show the colocalization of intracellular bacteria (red) with LBPA (green), and, in the upper left part of the merged images, the percentage of colocalization. Download FIG S2, TIF file, 2.0 MB. Copyright © 2023 Spera et al. 2023 Spera et al. https://creativecommons.org/licenses/by/4.0/ This content is distributed under the terms of the Creative Commons Attribution 4.0 International license.

10.1128/mbio.03338-22.3 FIG S3 The drug manipulation of MVBs affects the egress of Brucella from infected J774 A.1 cells. J774 A.1 cells were infected with B. abortus and were treated or not with the MVBs inhibitors (A) 15 nM DMA, (B) 5 μM GW4869, or (C) the MVBs promoter 7 μM monensin. Intracellular and extracellular bacteria were quantified by direct plating on TSB agar plates. Download FIG S3, TIF file, 1.8 MB. Copyright © 2023 Spera et al. 2023 Spera et al. https://creativecommons.org/licenses/by/4.0/ This content is distributed under the terms of the Creative Commons Attribution 4.0 International license.

**FIG 5:**
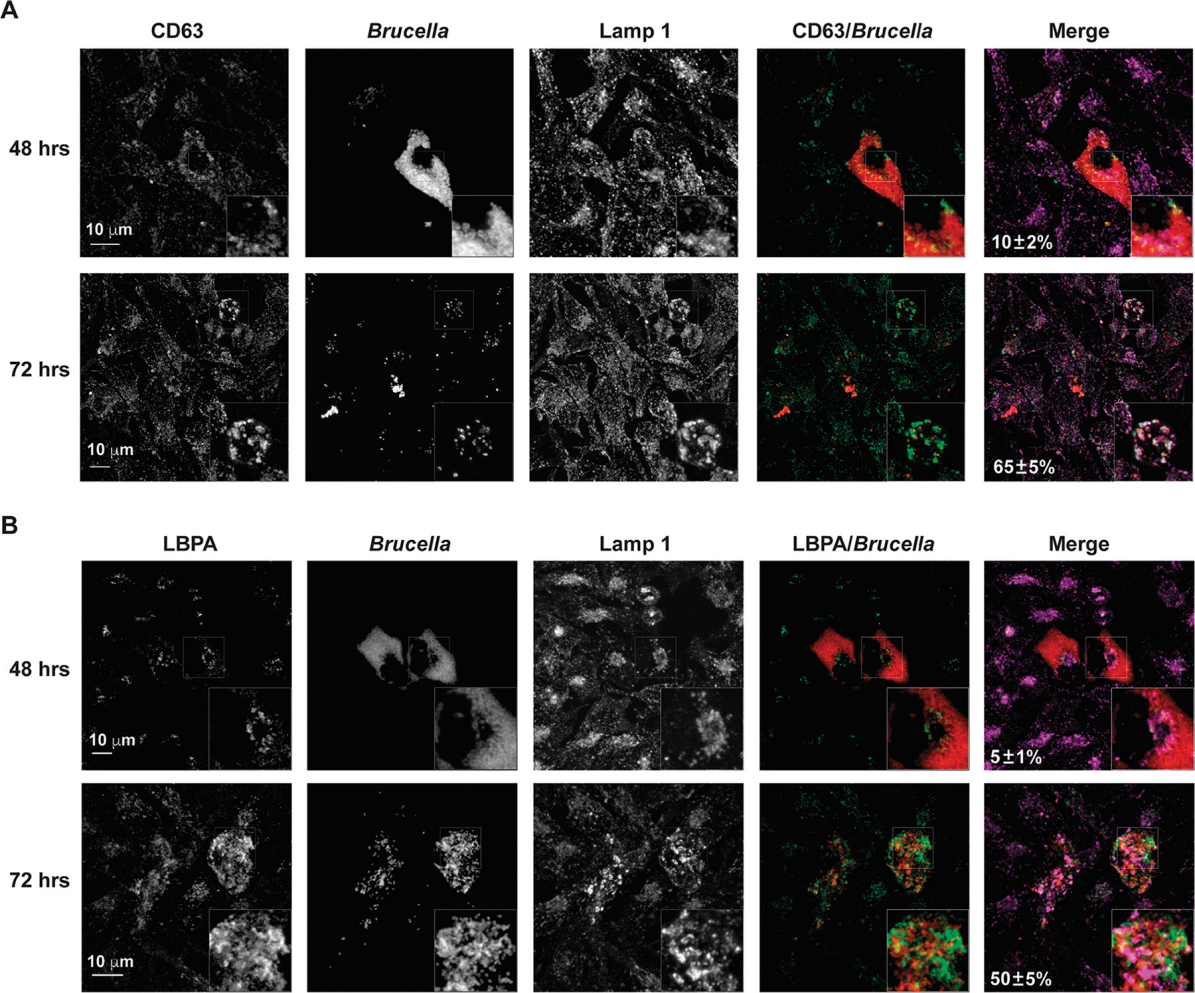
Colocalization of LAMP-1 with Brucella and LBPA or CD63 in infected cells. HeLa cells were infected with B. abortus DsRed and were subjected to immunofluorescence staining. This was followed by confocal microscopy, as described in Materials and Methods. The images show the staining and colocalization of Brucella (red), LAMP-1 (purple), and CD63 (A) or LBPA (green) (B) at 48 and 72 h postinfection.

It has been previously reported that, for the completion of the intracellular life cycle, Brucella hijacks autophagy to exit the host cell and promote a new round of infection. In this report, the authors found that one of the markers that Brucella acquires for this last phase of the intracellular life cycle is LAMP-1 (13), calling this vacuole the autophagic Brucella-containing vacuole (aBCV). Since our results indicate that Brucella also exploits MVBs for the egress process, two hypotheses arise: (i) both aBCV and MVB processes are part of the same pathway in a simultaneous or consecutive manner, or (ii) Brucella exploits at least two different mechanisms for egress. To analyze these possibilities, we infected HeLa cells. At 72 h postinfection, we determined the colocalization of Brucella with LAMP-1 and CD63 as well as that of Brucella with LAMP-1 and LBPA. As can be observed in Fig. 5A and B, a significant number of bacteria colocalized with LAMP-1 and CD63 (65 ± 5%) or with LAMP-1 and LBPA (50 ± 5%), strongly suggesting that the described aBCV (13) could be part of the MVB pathway that is described by this report (see Discussion).

### Contribution of the egress to the infection rate of Brucella.

**FIG 6:**
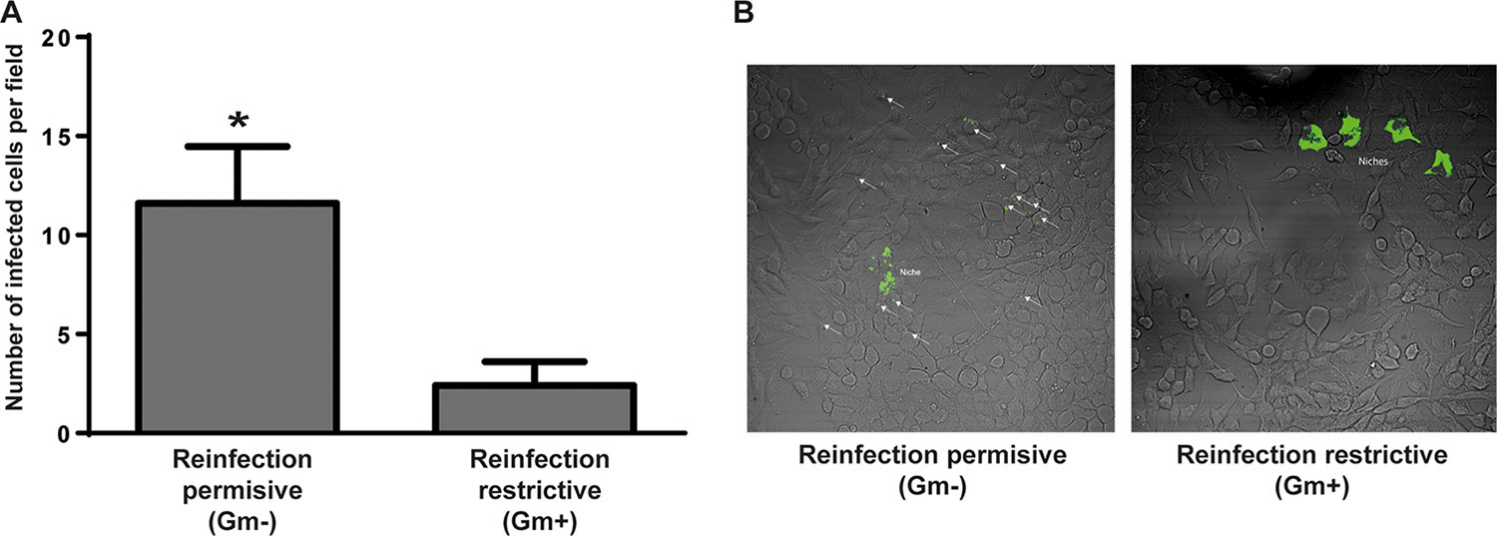
Brucella egress contributes to the infection process *in vitro*. Cell infections were carried out in the presence of gentamicin (reinfection restrictive condition) or absence of gentamicin (reinfection permissive conditions). At 48 h postinfection, the number of infected cells was determined by immunofluorescence with an anti-Brucella antibody. (A) Quantification of infected cells per field (*n* = 10). (B) The images are representative and show cells with bacterial niches (from the original infection event) and reinfected cells with only a few bacteria (arrows).

Even though it has been demonstrated by others and in this manuscript that the last phase of the intracellular life cycle of Brucella is the programmed egress, the contribution of this event to the pathogenesis of the infection has never been determined. Particularly, due to the nature of the antibiotic protection assay, it is not possible to measure the contributions of the egress and the reinfection to the virulence efficiency. To determine this contribution, we carried out infections of HeLa cells in the presence or absence of extracellular antibiotics. For this, cells were infected with Brucella. After 1 h, antibiotics were added for another hour to kill the remaining extracellular bacteria. At this point, we either kept the gentamicin for the rest of the assay (standard antibiotic protection assay) or removed it to allow for reinfection by the egressed bacteria. At 48 h postinfection, we determined by immunofluorescence the number of infected cells per field that contained 1 or more bacteria. As can be observed in Fig. 6, the absence of antibiotics showed that many cells that neighbored cells with niches (initially infected) were infected with 1 to 3 bacteria (reinfected), thereby resulting in a 350% increase in the number of infected cells. Similar results were observed in J774 A.1 cells (Fig. S4). Our data indicate that the processes of egress and reinfection by Brucella have a significant impact on the infection efficiency of cultured cells.

10.1128/mbio.03338-22.4 FIG S4 Brucella egress contributes to the infection process *in vitro*. Infections of J774 A.1 cells were carried out either in the presence of gentamicin (reinfection restrictive condition) or in the absence of gentamicin (reinfection permissive condition). At 48 hrs postinfection, the number of infected cells was determined by immunofluorescence with an anti-Brucella antibody. (A) Quantification of infected cells per field (*n* = 10). (B) The images are representative and show cells with bacterial niches (from the original infection event) and reinfected cells with only a few bacteria. Download FIG S4, TIF file, 1.9 MB. Copyright © 2023 Spera et al. 2023 Spera et al. https://creativecommons.org/licenses/by/4.0/ This content is distributed under the terms of the Creative Commons Attribution 4.0 International license.

## DISCUSSION

Intracellular pathogens have complex life cycles, as they must invade, survive, replicate in their host cells in many cases, and eventually egress to initiate a new infectious cycle. This last stage, egress, has been poorly studied in intracellular pathogens, even though it is central, as it might determine the efficiency of a new cycle or the induction of the host immune response that could, eventually, control the infection. Egress can occur by two different mechanisms (30): it can be lytic, a process that implies host cell death and a proinflammatory response, or nonlytic, in which the egress preserves the integrity of the cell and the immune response is mainly anti-inflammatory. These latter type of mechanism is generally called “vomocytosis”, and it depends on the interplay between the pathogen and host-cell factors, such as actin polymerization, microtubule modulation, phagosomal pH, inflammation, and exocytosis signals, among others (1–3). Several mechanisms have been described for this type of “silent” egress, such as the ones utilized by Cryptococcus neoformans (31), Legionella pneumophila (32, 33), and Mycobacterium tuberculosis (34).

Brucella is a well-adapted intracellular pathogen with the capacity to establish chronic infections, even in the presence of a robust immune response (5). This type of infectious strategy implies many cycles of infection-egress-infection that must necessarily occur silently in order to avoid the inflammatory immune response. Like the other virulence mechanisms that Brucella has evolved, this is also part of the stealthy strategy that this bacterium deploys for pathogenesis.

In the manuscript, we further study and characterize the egress of Brucella abortus from host cells. We found that the process of egress is massively triggered between 48 and 72 h postinfection and that the extracellular bacteria outnumber the intracellular population, an indication that the process is highly regulated and is probably exploited by the bacterium. For the first time, we monitored by live video microscopy the egress of Brucella from infected cells. Our results showed that between 48 and 72 h postinfection, B. abortus reorganizes the replicative niche and enters an egression mode. We also found that the bacteria exit the cells mostly in groups that either remain in the extracellular space or, in other cases, infect a neighboring cell. To our knowledge, this is the first report that shows, in real time, the egress of B. abortus from a host cell. During the live microscopy experiments, we noticed that many egressing bacteria seemed to be surrounded by a membrane. To determine whether at least a subpopulation of the egressing bacteria are enclosed in a host membrane, we treated the extracellular bacteria that were present in the culture supernatant with gentamicin or with gentamicin and Triton X-100, a detergent that dissolves eucaryotic membranes. Our results indicate that a portion of the egressing bacteria are enclosed in a membrane that protects them from the antibiotic. To gain further insight into this process, we analyzed the presence of multivesicular body markers in the egressing bacteria. We found that between 48 and 72 h postinfection, a significant percentage of the intracellular bacteria acquires two MVB markers: CD63 and LBPA. Interestingly, we found extracellular bacteria that were also labeled with these markers, raising the possibility that these structures might play a role in the reinfection of new cells. Moreover, when we determined the colocalization of both markers with the aBCV marker LAMP-1 at 72 h postinfection, we found that a significant proportion of them were double-labeled, strongly suggesting that the two pathways are either connected or are the same one. Consistent with the finding that egressing bacteria acquire MVB features, infection assays in the presence of two different inhibitors and a promoter of MVBs showed that this pathway contributes to this final stage of the intracellular cycle. Recently, Altamirano-Silva et al. (35) demonstrated that an intracellular passage of Brucella increases the infection efficiency in a subsequent intracellular infectious round. Even though we do not have evidence to support the idea that the egressing bacteria are more infective, it is tempting to speculate that the observation of Altamirano-Silva et al. and the observation of Brucella egressing in MVBs could be connected. The hijacking of MVBs by different intracellular pathogens has been described. Uropathogenic Escherichia coli (UPEC) has been observed to traffic in MVB to fuse with lysosomes. The pathogen manipulates this organelle to exit from infected bladder epithelial cells. The drug inhibition of MVBs in infected cells resulted in a reduction of bacterial expulsion (17). Chlamydia trachomatis is an obligated intracellular bacterial pathogen that replicates in a unique, membrane-bound vacuole, termed an inclusion body. Although the effects of MVBs on bacterial egress have not been studied, the recruitment of MVBs biosynthetic precursors results essential for inclusion maturation and intracellular bacterial growth (22, 23).

It has been described that most intracellular pathogens utilize more than one exit strategy, dependent on the life cycle stage, environmental factors, and host cell type (3). The only report that has shed some light on the egression mechanism of Brucella proposed that the process exploits autophagy (13). The authors described that between 48 and 72 h postinfection, the BCV acquires LAMP-1, as well as autophagic features, and that this vacuole, which the authors named autophagic BCV (aBCV), contributes to the formation of infection foci that neighbor the infected cells.

Our results indicate that a significant proportion of the BCVs that egress from the infected cell hijack the MVB pathway to reach the extracellular space and infect new cells. This raises the question of whether Brucella is egressing from infected cells in autophagic-like BCVs or in MVB-like BCVs. Our evidence shows that a proportion of these BCVs are also labeled with LAMP-1, indicating that Brucella-egressing-vacuoles have both autophagic and MVB features. This raises the question of whether there is a link between autophagy and MVBs. It has been described that autophagosomes can fuse with MVBs to form vesicles called amphisomes (36, 37). Amphisomes can either fuse with lysosomes to form autolysosomes and degrade their content or fuse to the plasma membrane and release their content into the extracellular space, thereby linking autophagy with our observations. One hypothesis could be that the pathway described by us and the one identified in (13) are part of the same process and could be acting sequentially. We propose that after the intracellular replication phase, the replicative niche disassembles, and the BCVs acquire autophagic features, eventually fuse with the MVB pathway to form amphisomes, and finally fuse with the plasma membrane for bacterial egress.

## MATERIALS AND METHODS

### Media and culture conditions.

Brucella abortus (14) and its derivative strains were grown at 37°C in tryptic soy broth (TSB). If necessary, medium was supplemented with the appropriate antibiotics at the indicated final concentrations: ampicillin, 100 μg/mL and nalidixic acid, 5 μg/mL. HeLa and J774 A.1 cells were maintained in RPMI medium (Invitrogen) containing 5% fetal bovine serum (FBS) at 37°C in 5% CO_2_. Bone marrow-derived macrophages (BMDM) were isolated from the femurs of 6 to 10-week-old C57BL/6 female mice and were differentiated into macrophages as described (15). The manipulation of B. abortus was performed at the biosafety level 3 (BSL3) laboratory facility at the Universidad Nacional de San Martín. The animal procedures were approved by the local regulatory agencies (CICUAE-UNSAM).

### Cell infections, bacterial egress quantification, and extracellular gentamicin protection assays.

Antibiotic protection assays were performed in HeLa, J774 A.1, or BMDM as described in (16). Cells were seeded in 24-well plates in RPMI medium at 10^5^ cells/mL. The cells were incubated overnight at 37°C. Brucella abortus 2308 was grown in TSB with the appropriate antibiotics for 24 h and was diluted in culture medium prior to infection. The bacterial suspension was added at different multiplicities of infection (1:1,000 for HeLa and 1:500 for J774 A.1 and BMDM) and centrifuged at 300 g for 10 min. After 1 h of incubation at 37°C, the cells were washed, and fresh medium containing 100 μg/mL of streptomycin and 50 μg/mL of gentamicin was added for 1 h to kill the uninternalized bacteria. Then, the cells were washed 3 times with PBS, and the medium was replaced with medium either containing 25 μg/mL gentamicin (for the reinfection restrictive conditions) or without antibiotics (for the reinfection permissive conditions). For the intracellular bacterial quantification at 24, 48, and 72 h postinfection, the cells were washed and lysed with 0.1% Triton X-100, and colony forming units (CFU) were determined by direct plating on TSB agar plates. For the bacterial egress quantification, the supernatants of the infected cells were processed as described in (17). The supernatants were collected in 24 h periods to minimize the extracellular replication effect (0 to 24 h, 24 to 48 h, and 48 to 72 h) and were centrifuged 3 times to remove detached or dead cells and debris (200 × *g* for 5 min, 200 × *g* for 5 min and, 310 × *g* for 5 min). The CFU were determined by direct plating on TSB agar plates. Extracellular gentamicin protection assays were performed as described in (17). The supernatants were centrifuged 3 times as described above, treated for 1 h with 50 μg/mL gentamicin in the presence or absence of Triton X-100 0.1%, and washed with PBS. The CFU were determined by direct plating on TSB agar.

### Cell transfection, live cell imaging, and immunofluorescence assays.

For the live cell imaging, HeLa cells were seeded on 35 mm dishes with 20 mm glass-like polymer bottom wells (Cellvis) at 4 × 10^5^ cells per well and infected as described above with B. abortus expressing DsRed (2308-DsRed). At 48 h postinfection, the cells were washed 3 times with sterile PBS. The medium was replaced to remove antibiotics, and the dishes were placed in a 37°C, CO_2_-buffered outer environmental chamber for live cell imaging. For the visualization of the colocalization of extracellular Brucella with CD63, the HeLa cells were transfected with pCMV-Sport6-CD63-pHluorin (AddGene) using Lipofectamine 3000 (Invitrogen), according to the manufacturer’s instructions, at 24 h prior to infection. Images of bacterial egress and of the extracellular colocalization of Brucella*-*DsRed with CD63 were acquired between 48 to 72 h postinfection by using an inverted microscope Zeiss Axio Observer 7 with an attached Colibri R(G/Y) B-UV LED-based illumination source and a thermal stable chamber at 37°C (lab-made). The images were taken with a Zeiss ZEN 2.3 Pro and an oil 40× Plan APO 1,4NA objective. The postacquisition analysis was conducted using the ImageJ 1.51p software package.

For the intracellular colocalization studies, all incubations were performed under cellular permeabilizing conditions. 4% paraformaldehyde-fixed cells were washed twice, and coverslips were incubated in blocking buffer (PBS with 10% horse serum and 0.1% saponin) containing primary antibodies for 30 min, except for anti-LBPA, which was incubated overnight. After two washes in PBS, the coverslips were incubated for 30 min in blocking buffer containing secondary antibodies. Finally, the coverslips were washed three times in PBS and once in Milli-Q water and were mounted on glass slides using Fluorsave (Calbiochem). The primary antibodies used were mouse anti-CD63 (Sigma) at 1:500, mouse anti-LBPA (Echelon Biosciences) at 1:100, and rabbit anti-Lamp1 at 1:500 (Sigma). The secondary antibodies used were Alexa Fluor 568 goat anti-mouse IgG at 1:4,000, Alexa Fluor 488 anti-mouse IgG at 1:4,000, and Alexa Fluor 647 anti-rabbit IgG at 1:4,000 (Molecular Probes, Invitrogen). Confocal images were acquired using an IX-81 microscope attached to a FV-1000 confocal module with a Plan APO 60, 1.42-numerical-aperture (NA) oil immersion objective (Olympus, Japan). The acquisition software used was FV 10-ASW 3.1. The images were treated using ImageJ 1.45s software (NIH, USA). For quantifying the colocalization between Brucella and the CD63, LAMP1, and LBPA markers, 10 pictures were randomly taken, and all of the bacteria per field were analyzed. The infection-staining and the quantifications were performed by different people to avoid bias.

### Effects of drugs on bacterial egress.

HeLa cells were infected with B. abortus 2308 as described above. At different times postinfection, the cells were washed with PBS to remove antibiotics and were treated with either 5 μM GW4869 (Sigma), 15 nM 5-(N,N dimethyl) amiloride (DMA) (Sigma), or 7 μM monensin (Sigma) or with RPMI as a control (or DMSO in cases of a DMA control). Monensin was added at 48 h postinfection for 3 h, and the bacterial egress was measured between 48 and 51 h postinfection. DMA was added 2 h postinfection, and extracellular bacteria were collected between 24 and 48 h of infection. GW4869 was added at 24 h postinfection for 48 h, and the bacterial egress was measured between 48 and 72 h.

### Statistical analysis.

The statistical analyses were performed using GraphPad Prism v.6 (GraphPad Software, La Jolla, CA, USA). The differences between the groups were calculated via the Student’s *t* test for the normally distributed variables via the nonparametric Mann-Whitney test for the nonnormally distributed variables. A *P* value of <0.05 (*) or <0.01 (**) was considered to be indicative of a statistically significant result.

10.1128/mbio.03338-22.5 VIDEO S1 Brucella abortus exits infected cells in vesicles. Movie from the time-lapse video microscopy, showing B. abortus DsRed (green) egressing from infected HeLa cells (DIC). The arrows indicate a cluster of bacteria egressing in a vesicle-like structure and another cluster egressing from an infected cell and invading the neighboring one. The selected video of the merged DIC and the fluorescence images are representative of the images observed between 48 and 72 hrs postinfection. T = 0 corresponds to 52 hrs postinfection. Bars: 10 μm. Download Movie S1, MOV file, 1.8 MB. Copyright © 2023 Spera et al. 2023 Spera et al. https://creativecommons.org/licenses/by/4.0/ This content is distributed under the terms of the Creative Commons Attribution 4.0 International license.

## References

[1] Friedrich N, Hagedorn M, Soldati-Favre D, Soldati T. 2012. Prison break: pathogens' strategies to egress from host cells. Microbiol Mol Biol Rev 76:707–720. doi:10.1128/MMBR.00024-12. 23204363 PMC3510522

[2] Traven A, Naderer T. 2014. Microbial egress: a hitchhiker's guide to freedom. PLoS Pathog 10:e1004201. doi:10.1371/journal.ppat.1004201. 25057992 PMC4110034

[3] Flieger A, Frischknecht F, Hacker G, Hornef MW, Pradel G. 2018. Pathways of host cell exit by intracellular pathogens. Microb Cell 5:525–544. doi:10.15698/mic2018.12.659. 30533418 PMC6282021

[4] Corbel MJ. 1997. Brucellosis: an overview. Emerg Infect Dis 3:213–221. doi:10.3201/eid0302.970219. 9204307 PMC2627605

[5] Byndloss MX, Tsolis RM. 2016. Brucella spp. virulence factors and immunity. Annu Rev Anim Biosci 4:111–127. doi:10.1146/annurev-animal-021815-111326. 26734887

[6] Bialer MG, Sycz G, Munoz Gonzalez F, Ferrero MC, Baldi PC, Zorreguieta A. 2020. Adhesins of Brucella: their roles in the interaction with the host. Pathogens 9:942. doi:10.3390/pathogens9110942. 33198223 PMC7697752

[7] Czibener C, Merwaiss F, Guaimas F, Del Giudice MG, Serantes DA, Spera JM, Ugalde JE. 2016. BigA is a novel adhesin of Brucella that mediates adhesion to epithelial cells. Cell Microbiol 18:500–513. doi:10.1111/cmi.12526. 26400021

[8] Lopez P, Guaimas F, Czibener C, Ugalde JE. 2020. A genomic island in Brucella involved in the adhesion to host cells: identification of a new adhesin and a translocation factor. Cell Microbiol 22:e13245. 32657513 10.1111/cmi.13245

[9] Pizarro-Cerda J, Meresse S, Parton RG, van der Goot G, Sola-Landa A, Lopez-Goni I, Moreno E, Gorvel JP. 1998. Brucella abortus transits through the autophagic pathway and replicates in the endoplasmic reticulum of nonprofessional phagocytes. Infect Immun 66:5711–5724. doi:10.1128/IAI.66.12.5711-5724.1998. 9826346 PMC108722

[10] Comerci DJ, Martinez-Lorenzo MJ, Sieira R, Gorvel JP, Ugalde RA. 2001. Essential role of the VirB machinery in the maturation of the Brucella abortus-containing vacuole. Cell Microbiol 3:159–168. doi:10.1046/j.1462-5822.2001.00102.x. 11260139

[11] Celli J, de Chastellier C, Franchini DM, Pizarro-Cerda J, Moreno E, Gorvel JP. 2003. Brucella evades macrophage killing via VirB-dependent sustained interactions with the endoplasmic reticulum. J Exp Med 198:545–556. doi:10.1084/jem.20030088. 12925673 PMC2194179

[12] Celli J. 2019. The intracellular life cycle of Brucella spp. Microbiol Spectr 7. doi:10.1128/microbiolspec.BAI-0006-2019. PMC6448592 30848234

[13] Starr T, Child R, Wehrly TD, Hansen B, Hwang S, Lopez-Otin C, Virgin HW, Celli J. 2012. Selective subversion of autophagy complexes facilitates completion of the Brucella intracellular cycle. Cell Host Microbe 11:33–45. doi:10.1016/j.chom.2011.12.002. 22264511 PMC3266535

[14] Chain PS, Comerci DJ, Tolmasky ME, Larimer FW, Malfatti SA, Vergez LM, Aguero F, Land ML, Ugalde RA, Garcia E. 2005. Whole-genome analyses of speciation events in pathogenic Brucellae. Infect Immun 73:8353–8361. doi:10.1128/IAI.73.12.8353-8361.2005. 16299333 PMC1307078

[15] Celli J, Salcedo SP, Gorvel JP. 2005. Brucella coopts the small GTPase Sar1 for intracellular replication. Proc Natl Acad Sci USA 102:1673–1678. doi:10.1073/pnas.0406873102. 15632218 PMC547823

[16] Ugalde JE, Czibener C, Feldman MF, Ugalde RA. 2000. Identification and characterization of the Brucella abortus phosphoglucomutase gene: role of lipopolysaccharide in virulence and intracellular multiplication. Infect Immun 68:5716–5723. doi:10.1128/IAI.68.10.5716-5723.2000. 10992476 PMC101528

[17] Miao Y, Li G, Zhang X, Xu H, Abraham SN. 2015. A TRP channel senses lysosome neutralization by pathogens to trigger their expulsion. Cell 161:1306–1319. doi:10.1016/j.cell.2015.05.009. 26027738 PMC4458218

[18] Badley RA, Martin WG, Schneider H. 1973. Dynamic behavior of fluorescent probes in lipid bilayer model membranes. Biochemistry 12:268–275. doi:10.1021/bi00726a015. 4683001

[19] Piper RC, Katzmann DJ. 2007. Biogenesis and function of multivesicular bodies. Annu Rev Cell Dev Biol 23:519–547. doi:10.1146/annurev.cellbio.23.090506.123319. 17506697 PMC2911632

[20] Hyenne V, Labouesse M, Goetz JG. 2018. The small GTPase Ral orchestrates MVB biogenesis and exosome secretion. Small GTPases 9:445–451. doi:10.1080/21541248.2016.1251378. 27875100 PMC6204988

[21] Ostrowski M, Carmo NB, Krumeich S, Fanget I, Raposo G, Savina A, Moita CF, Schauer K, Hume AN, Freitas RP, Goud B, Benaroch P, Hacohen N, Fukuda M, Desnos C, Seabra MC, Darchen F, Amigorena S, Moita LF, Thery C. 2010. Rab27a and Rab27b control different steps of the exosome secretion pathway. Nat Cell Biol 12:19–30. Sup pp 1–13. doi:10.1038/ncb2000. 19966785

[22] Beatty WL. 2008. Late endocytic multivesicular bodies intersect the chlamydial inclusion in the absence of CD63. Infect Immun 76:2872–2881. doi:10.1128/IAI.00129-08. 18426873 PMC2446703

[23] Beatty WL. 2006. Trafficking from CD63-positive late endocytic multivesicular bodies is essential for intracellular development of Chlamydia trachomatis. J Cell Sci 119:350–359. doi:10.1242/jcs.02733. 16410552

[24] Gruenberg J. 2020. Life in the lumen: the multivesicular endosome. Traffic 21:76–93. doi:10.1111/tra.12715. 31854087 PMC7004041

[25] Bissig C, Gruenberg J. 2013. Lipid sorting and multivesicular endosome biogenesis. Cold Spring Harb Perspect Biol 5:a016816. doi:10.1101/cshperspect.a016816. 24086044 PMC3783046

[26] Catalano M, O'Driscoll L. 2020. Inhibiting extracellular vesicles formation and release: a review of EV inhibitors. J Extracell Vesicles 9:1703244. doi:10.1080/20013078.2019.1703244. 32002167 PMC6968539

[27] Guo BB, Bellingham SA, Hill AF. 2016. Stimulating the release of exosomes increases the intercellular transfer of prions. J Biol Chem 291:5128–5137. doi:10.1074/jbc.M115.684258. 26769968 PMC4777847

[28] Savina A, Furlan M, Vidal M, Colombo MI. 2003. Exosome release is regulated by a calcium-dependent mechanism in K562 cells. J Biol Chem 278:20083–20090. doi:10.1074/jbc.M301642200. 12639953

[29] Hessvik NP, Llorente A. 2018. Current knowledge on exosome biogenesis and release. Cell Mol Life Sci 75:193–208. doi:10.1007/s00018-017-2595-9. 28733901 PMC5756260

[30] Hybiske K, Stephens RS. 2008. Exit strategies of intracellular pathogens. Nat Rev Microbiol 6:99–110. doi:10.1038/nrmicro1821. 18197167

[31] Johnston SA, May RC. 2013. Cryptococcus interactions with macrophages: evasion and manipulation of the phagosome by a fungal pathogen. Cell Microbiol 15:403–411. doi:10.1111/cmi.12067. 23127124

[32] Chen J, de Felipe KS, Clarke M, Lu H, Anderson OR, Segal G, Shuman HA. 2004. Legionella effectors that promote nonlytic release from protozoa. Science 303:1358–1361. doi:10.1126/science.1094226. 14988561

[33] Molmeret M, Abu Kwaik Y. 2002. How does Legionella pneumophila exit the host cell? Trends Microbiol 10:258–260. doi:10.1016/s0966-842x(02)02359-4. 12088652

[34] Hagedorn M, Rohde KH, Russell DG, Soldati T. 2009. Infection by tubercular mycobacteria is spread by nonlytic ejection from their amoeba hosts. Science 323:1729–1733. doi:10.1126/science.1169381. 19325115 PMC2770343

[35] Altamirano-Silva P, Cordero-Serrano M, Mendez-Montoya J, Chacon-Diaz C, Guzman-Verri C, Moreno E, Chaves-Olarte E. 2021. Intracellular passage triggers a molecular response in Brucella abortus that increases its infectiousness. Infect Immun 89:e0000421. doi:10.1128/IAI.00004-21. 33820813 PMC8373234

[36] Fader CM, Colombo MI. 2009. Autophagy and multivesicular bodies: two closely related partners. Cell Death Differ 16:70–78. doi:10.1038/cdd.2008.168. 19008921

[37] Ganesan D, Cai Q. 2021. Understanding amphisomes. Biochem J 478:1959–1976. doi:10.1042/BCJ20200917. 34047789 PMC8935502

