# Supplementary figures and images for: Brucella Egresses from Host Cells Exploiting Multivesicular Bodies
Source: mBio. 2023 Jan 9;14(1):e03338-22. doi: 10.1128/mbio.03338-22 (PMC9973279; doi:10.1128/mbio.03338-22)

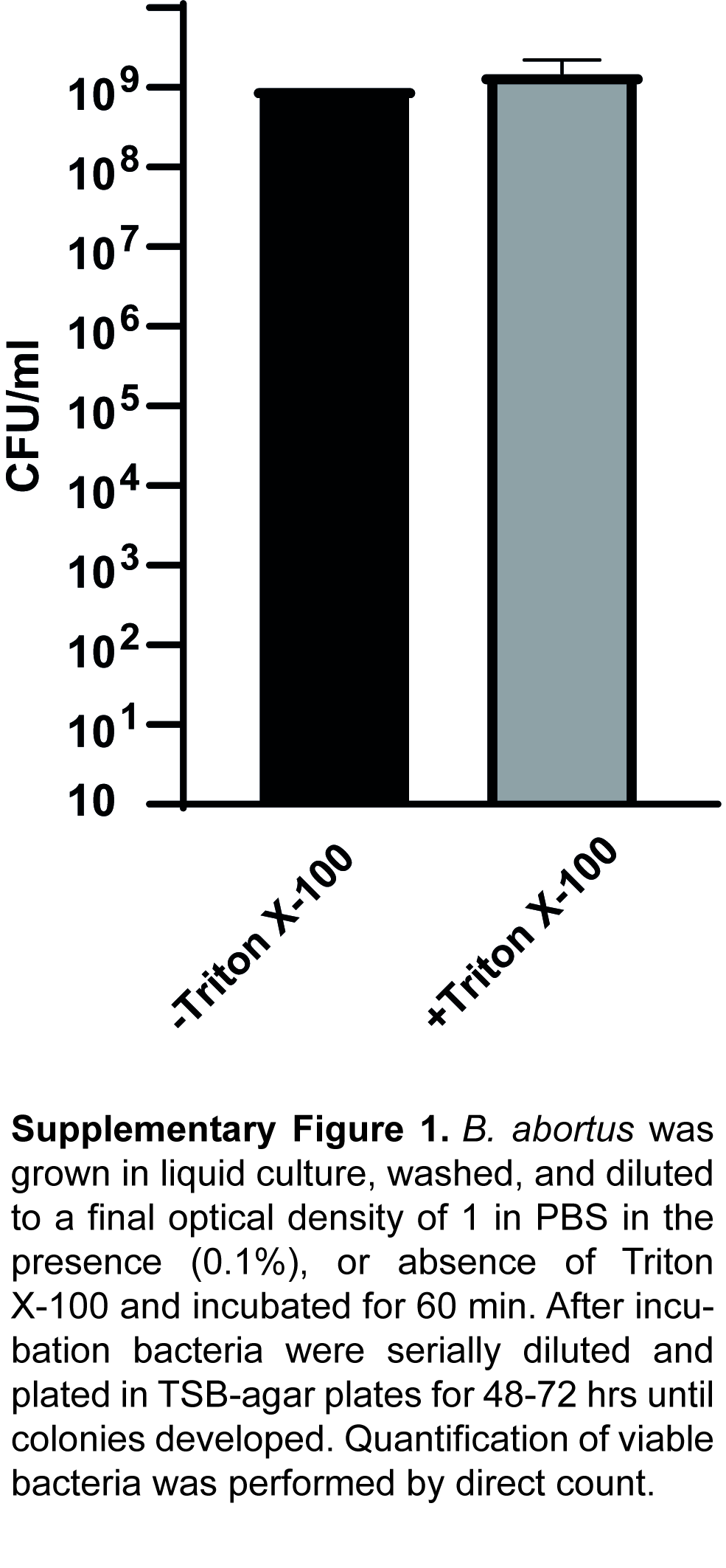

Supplement: FIG S1 [file mbio.03338-22-s0001.tif]

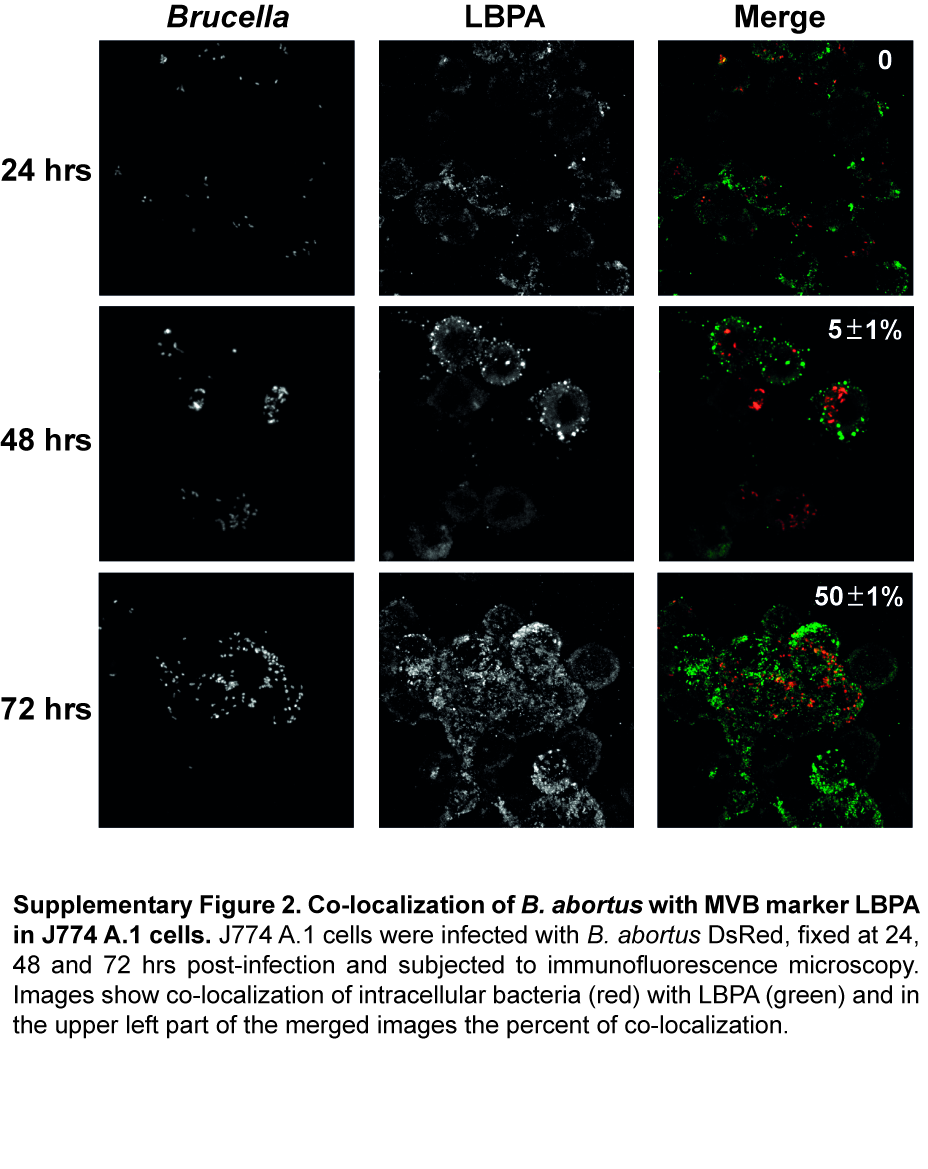

Supplement: FIG S2 [file mbio.03338-22-s0002.tif]

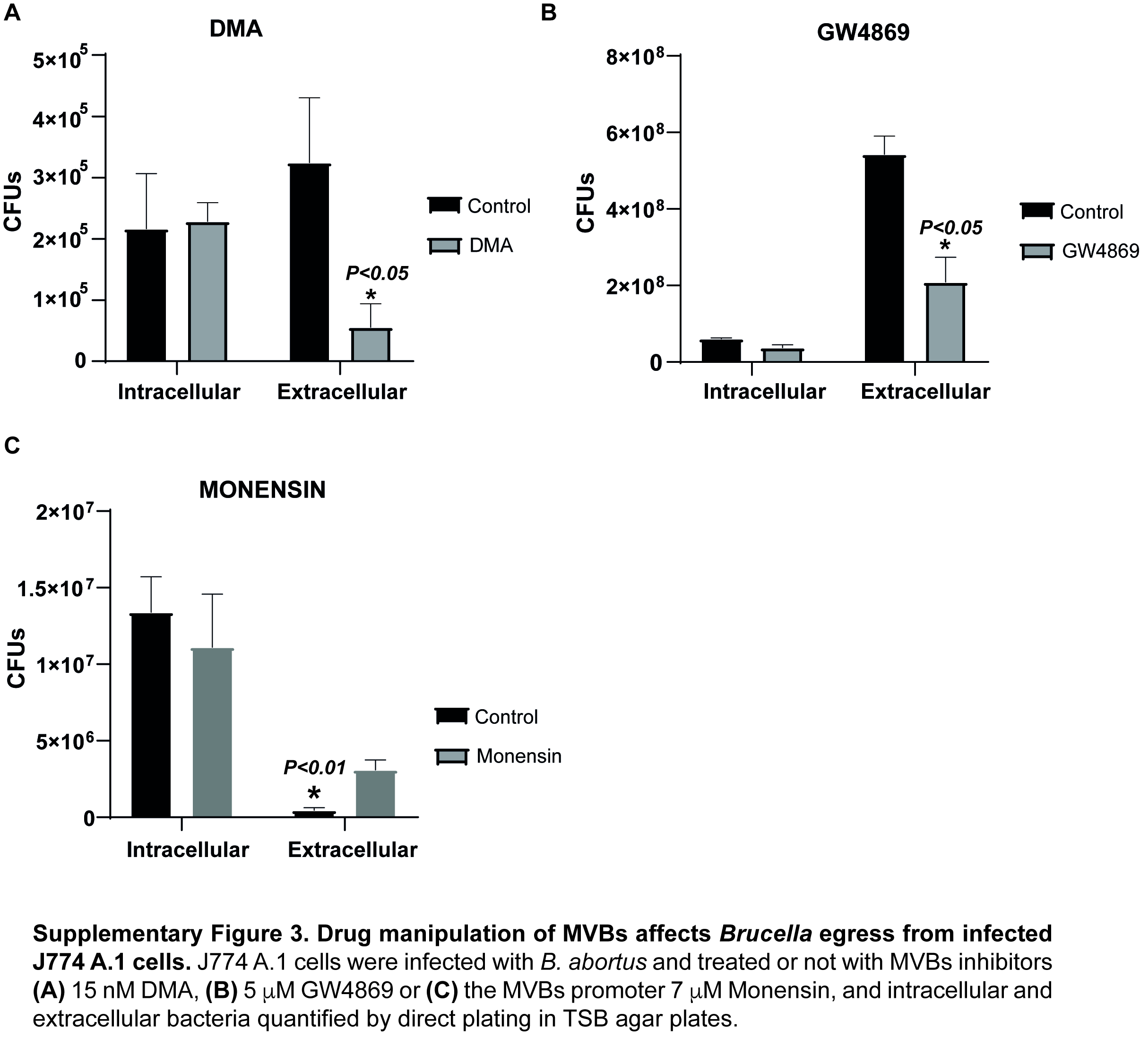

Supplement: FIG S3 [file mbio.03338-22-s0003.tif]

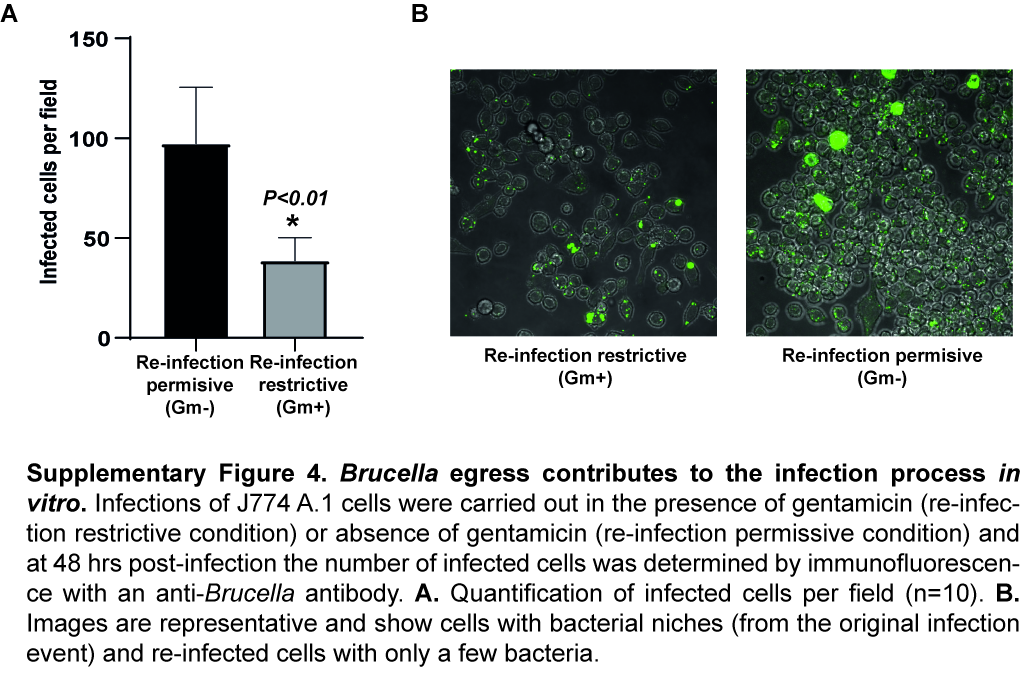

Supplement: FIG S4 [file mbio.03338-22-s0004.tif]
